# irAE-GPT: leveraging large language models to identify immune-related adverse events in electronic health records and clinical trial datasets

**DOI:** 10.1016/j.ebiom.2026.106227

**Published:** 2026-04-07

**Authors:** Cosmin A. Bejan, Michelle Wang, Sriram Venkateswaran, Ewa A. Bergmann, Laura Hiles, Yaomin Xu, G Scott Chandler, Sam Brondfield, Jordyn Silverstein, Francis Wright, Kimberly de Dios, Daniel M. Kim, Eric Mukherjee, Matthew S. Krantz, Lydia Yao, Douglas B. Johnson, Elizabeth J. Phillips, Justin M. Balko, Rajat Mohindra, Zoe Quandt

**Affiliations:** a Department of Biomedical Informatics, Vanderbilt University Medical Center, Nashville, USA; b Department of Bioengineering and Therapeutic Sciences, University of California San Francisco, San Francisco, USA; c Bakar Computational Health Sciences Institute, University of California San Francisco, San Francisco, USA; d F. Hoffmann-La Roche, Basel, Switzerland; e Roche Products Ltd, Welwyn Garden City, United Kingdom; f Department of Biostatistics, Vanderbilt University Medical Center, Nashville, USA; g Division of Hematology and Oncology, Department of Medicine, University of California San Francisco, San Francisco, USA; h Helen Diller Family Comprehensive Cancer Center, University of California San Francisco, San Francisco, USA; i Division of Hematology/Oncology, Department of Medicine, University of California, Los Angeles, USA; j Division of General Internal Medicine, School of Medicine, University of Colorado Anschutz Medical Campus, Aurora, USA; k Diabetes Center, University of California San Francisco, San Francisco, USA; l Division of Endocrinology and Metabolism, Department of Medicine, University of California San Francisco, San Francisco, USA; m Department of Medicine, Cedars-Sinai Medical Center, Los Angeles, USA; n Department of Dermatology, Vanderbilt University Medical Center, Nashville, USA; o Division of Allergy, Pulmonary, and Critical Care Medicine, Department of Medicine, Vanderbilt University Medical Center, Nashville, USA; p Department of Medicine, Vanderbilt University Medical Center, Nashville, USA; q Institute for Immunology and Infectious Diseases, Murdoch University, Murdoch, Australia

**Keywords:** Generative artificial intelligence, Large language models, Immune-related adverse events, Electronic health records, Clinical trials

## Abstract

**Background:**

Large language models (LLMs) have emerged as transformative technologies, revolutionising natural language understanding and generation across various domains, including medicine. In this study, we investigated the capabilities, limitations, and generalisability of Generative Pre-trained Transformer (GPT) models in analysing unstructured patient notes from large healthcare datasets to identify immune-related adverse events (irAEs) associated with the use of immune checkpoint inhibitor (ICI) therapy.

**Methods:**

We evaluated the performance of GPT-3.5, GPT-4, and GPT-4o models on manually annotated datasets of patients receiving ICI therapy, sampled from two electronic health record (EHR) systems and seven clinical trials. A zero-shot prompt was designed to exhaustively identify irAEs at both the patient level (main analysis) and the note level (secondary analysis). The LLM-based system followed a multi-label classification approach to identify any combination of irAEs associated with individual patients or clinical notes. System evaluation was conducted for each available irAE as well as for broader categories of irAEs classified at the organ level.

**Findings:**

Our analysis included 442 patients across three institutions. The most common irAEs manually identified in the patient datasets included pneumonitis (N = 64), colitis (N = 56), rash (N = 32), and hepatitis (N = 28). The GPT models demonstrated generalisable abilities in identifying irAEs across EHRs and clinical trial reports. Overall, the models achieved relatively high sensitivity and specificity but only moderate positive predictive values, reflecting a potential bias towards overpredicting irAE outcomes. GPT-4o achieved the highest F1 and micro-averaged F1 scores for both patient-level and note-level evaluations. Highest performance was observed in the haematological (F1 range = 1.0–1.0), gastrointestinal (F1 range = 0.81–0.85), and musculoskeletal and rheumatologic (F1 range = 0.67–1.0) irAE categories. Error analysis uncovered substantial limitations of GPT models in handling textual causation, where adverse events should not only be accurately identified in clinical text but also causally linked to immune checkpoint inhibitors.

**Interpretation:**

This study demonstrated that GPT models can automate the detection of immune related adverse events in varied healthcare datasets, reducing the burden on physicians and other healthcare professionals by limiting the need for manual review. This capability will accelerate the generation of safety insights across large healthcare datasets and facilitate the characterisation of patient-level drivers of toxicities, thus enhancing safety monitoring and ultimately improving patient care.

**Funding:**

National Institutes of Health, Roche, National Health and Medical Research Council of Australia, Stevens-Johnson Syndrome Foundation, Angela Anderson Research Fund, Larry L Hillblom Foundation and UCSF Research Allocation Program.

**Research in context**

**Evidence before this study**

We searched PubMed on February 27, 2026, without restrictions on language or date, using the following query (“irAE” OR “immune-related adverse events”) AND (“LLM” OR “GPT” OR “NLP” OR “large language models” OR “deep learning” OR “natural language processing” OR “machine learning” OR “artificial intelligence” OR “generative AI”). Out of 121 retrieved articles, we identified six studies that applied machine learning or natural language processing to detect immune-related adverse events (irAEs) in patient data. These studies focused on identifying one or more specific irAEs (up to four), or on predicting a binary outcome based on the presence of irAEs in patient records. However, none of them performed a thorough assessment of all irAEs present in a specific dataset. Further, no study has used clinical trial data for irAE identification. Three studies used clinical notes from electronic health record (EHR) databases, but none examined whether their models generalise across various clinical settings, such as outpatient and inpatient care. The methodologies proposed in the studies based on clinical notes include word embeddings, neural networks, a deep learning system with 110 million parameters leveraging the bidirectional encoder representations from transformers (BERT) architecture, and a lightweight generative artificial intelligence (GenAI) model with 7 billion parameters. The evaluation of these methodologies highlighted substantial room for improvement in irAE identification within unstructured patient notes, as well as an opportunity to explore the potential of the latest GenAI technologies, such as generative pre-trained transformer (GPT) models.

**Added value of this study**

In this multicenter study, we developed and validated a GPT-based model, irAE-GPT, to systematically identify irAEs in clinical notes from two large EHR systems and patient reports from seven clinical trials. We utilise GPT models, the most advanced GenAI technology available, to identify irAEs in clinical text. irAE-GPT showed robust performance and strong generalisation for irAE identification across diverse clinical text modalities, clinical settings, irAE types, and immune checkpoint inhibitor therapies, using data from multiple healthcare centers. These findings suggest that GPT models can automate irAE extraction across varied healthcare datasets, thereby reducing the reliance on labour-intensive manual annotation by specialised clinicians.

**Implications of all the available evidence**

By enabling scalable irAE case identification and decreasing dependence on clinician-led manual curation, irAE-GPT can support faster and more efficient implementation of irAE pharmacoepidemiologic research. Furthermore, the robust performance and generalisability demonstrated by irAE-GPT highlight its potential for deployment as a clinical decision support tool to enhance the safety and effectiveness of cancer immunotherapies and ultimately improve patient care.

## Introduction

While immune checkpoint inhibitors (ICIs) have greatly enhanced clinical outcomes across multiple cancer types,^1^, ^2^, ^3^ their use is often accompanied by immune-related adverse events (irAEs), which occur in 74–90% of patients depending on the type of ICI therapy.^4^ These events, which can be severe with combination immunotherapies, affect a broad spectrum of organs, including the colon, liver, lungs, heart, nervous system, skin, and endocrine system.^5^, ^6^, ^7^, ^8^ Optimising the management of irAEs in clinical practice, thereby increasing the safety and effectiveness of cancer immunotherapies, is a major focus in precision oncology research. However, current limitations on the timely and accurate identification of irAEs in clinical settings restrict the implementation of such studies at scale. Major challenges stem from the lack of generalisable methods for identifying all types of toxicities and the difficulties of analysing dynamically evolving real-world healthcare data (especially unstructured data) such as electronic health records (EHRs) and clinical trials datasets.

Previous work by our groups and others has shown that irAEs are poorly captured by structured EHR data like International Classification of Diseases (ICD) codes.^9^, ^10^, ^11^, ^12^ Moreover, specific ICD-10 codes for ICI associated irAEs have only become available recently^13^ and it would take time before they are routinely used to abstract irAE data from large healthcare datasets. In contrast, causal relationships between ICI exposures and their irAEs are better encoded in clinical notes, as they allow healthcare providers to document treatment plans, adverse drug reactions, and justifications for discontinuing medications in plain language and with increased detail.^11^^,^^12^^,^^14^ Still, the automatic identification of irAEs in clinical text poses significant technical challenges since the mention of an irAE in a patient note does not necessarily imply that the patient is currently experiencing the adverse event. For example, an irAE in clinical text may be marked as unlikely or possible, or presented hypothetically as a potential risk during ICI therapy. Further, even when such an event is positively asserted in clinical notes, its underlying cause may not be linked to an ICI exposure.

Recent progress of generative artificial intelligence (AI) technologies, such as Generative Pre-trained Transformer (GPT),^15^, ^16^, ^17^ underscores the potential of large language models (LLMs) as a transformative approach for identifying adverse events in patient notes.^18^^,^^19^ These advancements reflect a broader evolution in AI from traditional machine learning and earlier deep learning models to LLMs, which can learn rich language representations through large-scale pretraining and require minimal task-specific engineering or labelled data. Prompting alone enables LLMs to execute multiple tasks using zero-shot learning, resulting in significantly enhanced generalisation across diverse datasets and domains. In healthcare applications, these models have already shown exceptional proficiency in extracting linguistic patterns that convey complex clinical information including clinical phenotypes, assertion status, and relationships between medical concepts (e.g., drug–adverse reaction).^12^^,^^20^, ^21^, ^22^, ^23^, ^24^

This study describes the development and evaluation of irAE-GPT, a holistic system for high-throughput irAE identification in clinical text, leveraging state-of-the-art OpenAI GPT models−GPT-3.5, GPT-4, and GPT-4o−and two distinct data sources from multiple institutions: clinical trials and real-world EHR data. Clinical trial datasets summarise safety information–such as irAE symptoms, specific irAE diagnoses, and attributes like therapeutic exposure, treatment duration, and comorbidities–in semi-structured reports using standard terminology. In contrast, real-world EHR data capture a comprehensive, longitudinal record of patients’ health trajectories, encompassing diagnoses, procedures, treatments, medications, encounters, laboratory measurements, clinical notes, images, outcomes and other relevant clinical information over time. The objectives of this study were to: (1) assess the capabilities of GPT models for irAE identification in EHR and clinical trial datasets, (2) explore the limitations of GPT models for this task, and (3) evaluate the generalisability and reproducibility of these models across different data sources originating from multiple institutions.

## Methods

### Study design

This retrospective cohort study utilises data collected from two large EHR systems, Vanderbilt University Medical Center (VUMC) and University of California, San Francisco (UCSF), and from seven Roche-sponsored clinical trials.^25^, ^26^, ^27^, ^28^, ^29^, ^30^, ^31^ The study population included patients who received any of the following ICI therapies, either as monotherapy or in combination regimens: atezolizumab, avelumab, durvalumab, ipilimumab, nivolumab, or pembrolizumab. The main analysis was conducted at the patient level, where, for each patient in the study, irAE-GPT reviewed their corresponding notes to identify all possible irAEs resulting from the patient's exposure to ICI therapy. This analysis also involved the aggregation of irAE predictions by GPT models across all the notes of the same patient. A secondary analysis was carried out on VUMC data to assess irAE-GPT's performance in extracting irAEs caused by ICIs at the note level. As patients may experience none, one, or several irAEs following ICI exposure, a multi-label classification approach was adopted to ensure LLMs can identify any combination of irAEs associated with each patient or clinical note. System evaluation was conducted for each available irAE as well as for broader categories of irAEs classified at organ level. The irAEs corresponding to each irAE category are listed in Table S1. This study follows established guidelines for the transparent reporting of a multivariable model for individual prognosis or diagnosis (TRIPOD)-LLM (Table S2).^32^

### Ethics

This study was conducted in accordance with the Declaration of Helsinki ethical guidelines. Institutional review boards at VUMC (#161455) and UCSF (#17-22987) approved the study with a waiver of consent, and all required internal processes were followed to permit secondary use of data from seven Roche-sponsored trials (NCT02450331, NCT02031458, NCT02108652, NCT02486718, NCT02008227, NCT02302807 and NCT03024996). The study included only patients from the seven Roche-sponsored trials who consented to secondary use of their data use at enrolment. All records were de-identified prior to analysis.

### Data sources

In this study, expert clinical review was conducted for all EHR-identified irAEs, taking into account the time course, objective findings, and clinical symptoms to establish attribution. In the absence of a definitive diagnostic test for irAEs, clinical judgement by an experienced physician remains the standard approach. However, certain diagnoses such as hypothyroidism can often be confirmed based on typical laboratory abnormalities observed during ICI treatment (elevated TSH and low free T4), provided no alternative etiologies are present. Notably, each center dedicated substantial resources to manually reviewing patient records and annotating irAEs over the course of multiple years. Although multiple clinically trained staff contributed to the annotation process, complex or ambiguous cases were discussed and ultimately adjudicated by an experienced clinician.

At VUMC, the Synthetic Derivative, a de-identified EHR database of 4 million records, was used to sample patients who underwent ICI therapy between 2009 and 2019. Starting from the first day of ICI administration, the health record data associated with each patient was manually reviewed by clinical experts to label all irAEs experienced by the patient or ‘None’ in the event the patient had no adverse reactions caused by ICI therapy. For each patient, clinical notes timestamped between the first date of ICI exposure and up to six months after the last date of ICI administration were selected for LLM analysis. As the VUMC dataset covers irAEs from both inpatient and outpatient encounters regardless of severity, we decided to include all available note types to ensure that no potential true positives were missed, especially those documented in less commonly used notes. In addition to patient-level annotation of irAEs, the same labels (i.e., a list of irAEs or ‘None’) were used for note-level annotation on individual notes sampled from 20 patients of the above-mentioned dataset.

UCSF Clinical Data Warehouse (CDW), which contains de-identified EHR data captured after 2012 from approximately 6 million unique patients, was used to construct the UCSF cohort. The study team leveraged structured EHR data to identify 617 patients who were admitted to the hospital within 6 months of ICI exposure. Manual chart review narrowed this cohort to 114 subjects who were deemed definitely or probably admitted for hospitalisation due to at least one irAE.^33^ The clinical notes selected for this study included the admission, first and last history and physical (H&P) exam notes for that admission, first and last consult notes from various speciality departments for that admission, and discharge summaries for each hospitalisation. As hospitalisation typically reflects greater clinical severity, we expect the selected note types to contain comprehensive descriptions of the associated irAEs. Ultimately, the final UCSF cohort included a total of 70 patients with 74 hospital encounters, each having at least one of the specified types of clinical notes. Each patient was annotated with specific types of irAEs including the irAEs requiring admission and any other concurrent irAEs. Basic demographics and other baseline characteristics of each patient were extracted from structured EHR data (e.g., age, sex, race, etc.) and manual annotations (e.g., cancer types, cancer stage, etc.)

Clinical trials data came from seven completed Roche-sponsored clinical studies NCT02450331 (n = 809), NCT02031458 (n = 659), NCT02108652 (n = 429), NCT02486718 (n = 507), NCT02008227 (n = 613), NCT02302807 (n = 467) and NCT03024996 (n = 778) in genitourinary, renal and lung cancer indications (Figure S1). Adverse events in the ICI clinical trials were documented by site personnel in electronic Case Report Forms (eCRFs), in accordance with adverse event reporting requirements defined by the study protocols. Causal associations with ICI therapy were determined by reporting investigators based on a comprehensive review of each patient's data. irAEs were abstracted from narrative reports authored and reviewed by qualified physicians and scientists at the study sponsor drawing on data from the eCRFs and additional inputs from principal investigators or study sites. These events were extracted from the structured database using a broad search of MedDRA preferred terms and subsequently compared to results from the LLM analysis of free text narratives.

### Statistics

irAE-GPT is a high-throughput phenotyping system designed to systematically identify irAEs from EHR and clinical trial datasets. It leverages the Azure OpenAI Service, offering access to private, institutionally managed GPT model instances that ensure security and compliance with legal and business agreements. The GPT model instances evaluated in this study included GPT-3.5 (gpt-35-turbo-16k), GPT-4 (gpt-4-turbo), and GPT-4o.

**Fig. 1:**
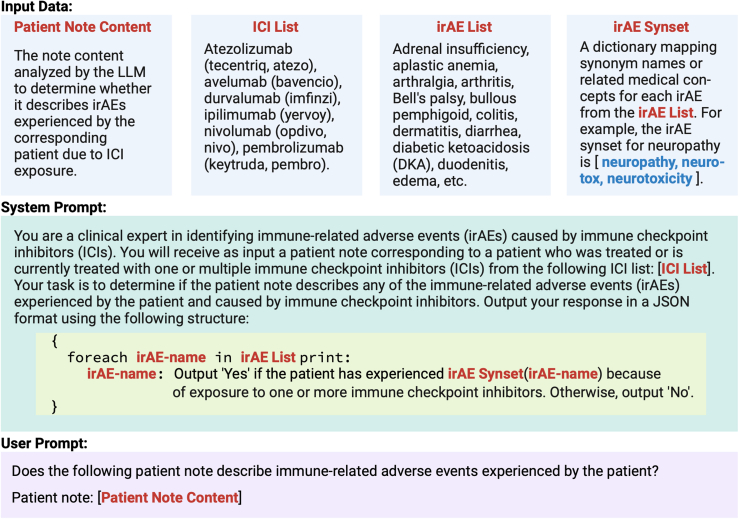
Prompt template for identifying irAEs due to ICI exposure in patient notes. The irAEs used for each dataset are listed in Tables S6–S8.

Central to the LLM-based system is a zero-shot prompt crafted to identify irAEs in a clinical note (Fig. 1). Specifically, for a given patient note, along with prespecified lists of irAEs and ICI treatments, the prompt systematically queries the LLM to determine whether the note describes the patient experiencing irAEs caused by exposure to any of their ICI treatments. For easier processing, the LLM output was required to be in JavaScript Object Notation (JSON) format, with each irAE corresponding to a binary response (‘Yes’ or ‘No’) based on its presence in the clinical note. Prompt optimisation was piloted using data from 30 patients with irAEs at VUMC, with several prompt formulations evaluated. The purpose of this pilot was to examine how minor changes in phrasing and formatting of natural language instructions affect performance, given prior evidence from our group and others that zero-shot LLM-based classifiers are highly sensitive to prompt construction.^21^^,^^34^, ^35^, ^36^ Enhanced performance was consistently observed when more detailed irAE descriptions were included, reflecting the varied terminology used to describe irAEs in clinical text. For this purpose, sets of text expressions describing synonyms, acronyms, or semantically related medical concepts–which we called “synsets”–were constructed for selected irAEs to better guide the LLM in the irAE identification process. For example, using the synset information corresponding to ‘*neuropathy’* listed in Table S3, its augmented query becomes “*Output ‘Yes’ if the patient has experienced neuropathy (neurotox, neurotoxicity) because of exposure to one or more immune checkpoint inhibitors. Otherwise, output ‘No’.*” This way, the LLM is explicitly informed that terms like ‘*neurotox*’ or ‘*neurotoxicity*’ may also be used to describe neuropathy in patient notes. Notably, the irAE list was adapted for each institution such that each element from the list was annotated at least once in the corresponding dataset. Similarly, multiple site-specific irAE synsets emerged due to variations in the manual annotation processes at each institution. For example, the prompt for the VUMC run had separate queries for colitis and diarrhoea whereas the UCSF prompt had only one query for both concepts because they were annotated under the same irAE label. In this case, the colitis synset for the UCSF prompt contained both ‘*colitis*’ and ‘*diarrhoea*’. The python code with the implementation of irAE-GPT is available at https://github.com/bejanlab/irAE-GPT.git.

### irAE-GPT evaluation

The system was deployed at each institution, and its evaluation utilised the complete dataset from the corresponding institution. The same zero-shot prompt and hyperparameters were used for each run except for site-specific irAE lists and synsets. Each LLM was executed with the temperature–a hyperparameter that controls the level of randomness or determinism in LLM output generation–set to 0. This setting is optimal for binary classification tasks, ensuring the model consistently selects the highest-probability token, corresponding to ‘*Yes*’ or ‘*No*’ for each irAE. Performance metrics including precision (positive predictive value or PPV), recall (sensitivity), specificity, and F1 score were computed by comparing the manual annotations against the results extracted by irAE-GPT for each irAE. Patient-level evaluation involved aggregating the LLM results from all notes per patient and comparing the aggregated results with the annotated irAE labels of the corresponding patient. To extract performance measures for irAE categories, we converted both irAE annotations and irAE results generated by the system using the mappings listed in Table S1. Micro- and macro-averaged results were also computed to assess the overall performance across all irAEs and irAE categories. Because our task emphasised the accurate identification of positive outcomes, F1 and micro-averaged F1 were selected as the primary evaluation metrics across all experiments, including the pilot study for prompt optimisation. Finally, error analysis was conducted based on manual assessment of clinical notes corresponding to false positive and false negative results.

### Role of funders

The funders had no role in the design of the study, collection and analysis of data, interpretation of the results, or preparation of the manuscript.

## Results

### Description of datasets

The cohorts at VUMC, UCSF, and Roche used for the main analysis included 100, 70, and 272 patients, respectively (Table 1). The mean age of the patients at ICI initiation across the three cohorts ranged from 62.8 to 65.2 years, with most being male and White. The VUMC and UCSF patients were more likely to be treated with a PD-(L)1 inhibitor followed by a combination of PD-(L)1 and CTLA-4 inhibitors while all Roche patients were treated with a PD-L1 inhibitor. Melanoma was the most common cancer type among VUMC and UCSF patients, while lung cancer was the most frequent in the selected Roche-sponsored clinical trials. Across datasets, the average number of notes per patient varied substantially, from 264.3 in the VUMC cohort to 1 in the Roche cohort. Token counts extracted with OpenAI's Tiktoken tokenizer (cl100k_base vocabulary) indicated that admission notes and discharge summaries were the longest documents in the VUMC dataset, while discharge summaries and H&P exam notes were longest in the UCSF dataset (Table S4). Similar trends in patient- and note-level characteristics emerged in the VUMC dataset (667 notes from 20 patients) used for the secondary analysis (Table S5).

**Table 1:** Selected characteristics of study participants across all datasets.

Characteristic	VUMC	UCSF	Roche
Patients, n	100	70	272
Notes, n (mean notes per patient)	26,432 (264.3)	487 (7.0)	272 (1.0)
Age at ICI initiation, mean years (SD^a^)	62.81 (11.7)	63 (13)	65.2 (NA)
Sex, n (%)			
Male	71 (71.0%)	42 (60%)	188 (69.1%)
Female	29 (29.0%)	28 (40%)	84 (30.9%)
Race/Ethnicity, n (%)			
White	95 (95.0%)	51 (73%)	NA
Black	3 (3.0%)	1 (1.4%)	NA
Asian	1 (1.0%)	6 (8.6%)	NA
Hispanic	1 (1.0%)	10 (14%)	NA
Other/Unknown	0 (0.0%)	2 (2.9%)	NA
Cancer, n (%)			
Lung	27 (27.0%)	10 (14%)	166 (61%)
Melanoma	36 (36.0%)	25 (36%)	0 (0.0%)
Renal	8 (8.0%)	8 (11%)	30 (11%)
Head and Neck	7 (7.0%)	5 (7.1%)	0 (0.0%)
Bladder	0 (0.0%)	1 (1.4%)	76 (28%)
Genitourinary	6 (6.0%)	0 (0.0%)	0 (0.0%)
Breast	0 (0.0%)	4 (5.7%)	0 (0.0%)
Hepatocellular	2 (2.0%)	0 (0.0%)	0 (0.0%)
MSI-H/dMMR Colon	1 (1.0%)	0 (0.0%)	0 (0.0%)
Gastric/Gastroesophogeal Junction	2 (2.0%)	3 (4.3%)	0 (0.0%)
Skin	0 (0.0%)	2 (2.9%)	0 (0.0%)
Other	7 (7.0%)	12 (17%)	0 (0.0%)
None	4 (4.0%)	0 (0.0%)	0 (0.0%)
ICI Type, n (%)			
Combination PD-(L)1 + CTLA-4 inhibitor	21 (21.0%)	25 (36%)	0 (0.0%)
PD-(L)1 inhibitor	68 (68.0%)	42 (60%)	272 (100%)
CTLA-4 inhibitor	11 (11.0%)	3 (4.3%)	0 (0.0%)
irAE Category, n (%)			
Cardiovascular	1 (1.0%)	4 (5.7%)	11 (4.0%)
Dermatologic	15 (15.0%)	8 (11%)	15 (5.5%)
Endocrine	15 (15.0%)	15 (21%)	21 (7.7%)
Gastrointestinal	17 (17.0%)	36 (51%)	42 (15.4%)
Haematological	1 (1.0%)	0 (0%)	4 (1.5%)
Musculoskeletal and Rheumatologic	10 (10.0%)	7 (10%)	1 (0.4%)
Neurologic	1 (1.0%)	5 (7.1%)	7 (2.6%)
Other	0 (0.0%)	4 (5.7%)	11 (4.0%)
Pulmonary	8 (8.0%)	17 (24%)	44 (16.2%)
Renal	2 (2.0%)	0 (0%)	4 (1.5%)
None	42 (42.0%)	0 (0%)	0 (0.0%)

a SD, standard deviation.

Each institute adopted its own strategy in annotating irAEs and selecting unstructured patient notes for LLM-based analysis. At VUMC, both the 100-patient cohort used for the main analysis and the 20-patient cohort used for the secondary analysis were randomly selected from a population of 747 patients who received ICI therapy and were manually annotated with irAE labels (Table S6). Since there were no restrictions imposed on the ICI exposed patients for irAE annotation, many patients were found as not experiencing any irAE (42 patients labelled as ‘None’ out of 100 patients selected for the study). From the patients with at least one irAE, most of them had rash (N = 10), colitis (N = 9), and pneumonitis (N = 8). As no restriction was imposed on patient note selection either (e.g., by note type), 26,432 of them (264 notes per patient, on average) were included for LLM-based irAE identification. At UCSF, all 70 patients were found as experiencing at least one irAE as they were selected for annotation from patients admitted for hospitalisation due to definite or probable irAE (hence, with more severe events). Gastrointestinal irAEs, mostly colitis (N = 23) and hepatitis (N = 15), and pneumonitis (N = 15) were the most common irAEs at hospitalisation (Table 1 and Table S7). For LLM-based irAE identification, the study team curated a set of 487 clinical notes from 74 hospital encounters (∼7 notes per patient) corresponding to the 70 patients. Finally, at Roche, there were 3471 patients who received ICI therapy in 7 completed trials. From the dataset, 272 patients, who corresponded to the same number of reports, were randomly extracted using a broad structured MedDRA search for possible irAEs. A subset of the most common irAEs were selected for the LLM-based analysis (Figure S1, Table S8). Notably, some adverse event terms extracted during this process may not qualify as irAEs (e.g., influenza) but might instead be indirectly associated with ICI therapy. For instance, influenza-like symptoms may be reported as influenza. In the Roche study cohort, the most common irAEs were pneumonitis (N = 41), colitis (N = 24), and rash (N = 15).

### Patient-level evaluation

**Fig. 2:**
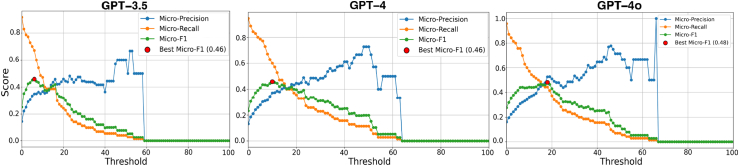
Trends of micro-averaged precision (Micro-Precision), recall (Micro-Recall), and F1 (Micro-F1) scores achieved by GPT models for various decision threshold values on the VUMC dataset.

We adopted simple strategies to aggregate the predictions for all the notes of the same patient. For VUMC results, we balanced the tradeoff between precision and recall by determining the optimal decision threshold—the number of notes with irAE-positive predictions—used to classify patient-level irAE outcomes. The optimal decision threshold values determined by GPT-3.5, GPT-4, and GPT-4o models for maximising the micro-averaged F1 score were 6, 10, and 18, respectively (Fig. 2). The patient-level irAE predictions on UCSF and Roche datasets were determined based on at least one positive prediction (decision threshold = 1) in any of the notes corresponding to the same patient.

Overall, the GPT models exhibited relatively high micro-averaged recall (VUMC: 0.57, UCSF: 0.86, Roche: 0.92) and specificity (VUMC: 0.97, UCSF: 0.92, Roche: 0.96) but achieved only moderate micro-averaged precision (VUMC: 0.42, UCSF: 0.41, Roche: 0.41) across all datasets (Tables S9–S11). For multiple irAEs at VUMC (e.g., haemolytic anaemia, lupus flare, bullous pemphigoid), UCSF (e.g., Stevens-Johnson syndrome, Guillain-Barre), and Roche (e.g., bladder tamponade, ascending flaccid paralysis), the GPT models achieved 100% F1 scores. However, the models' performance was relatively modest for several more common adverse events including arthritis (${F1}_{VUMC:GPT-4}=0.22$, ${F1}_{UCSF:GPT-3.5}=0.27$), rash (${F1}_{VUMC:GPT-3.5}=0.31$), colitis (${F1}_{VUMC:GPT-4o}=0.32$), fever (${F1}_{UCSF:GPT-4o}=0.32$), hypothyroidism (${F1}_{Roche:GPT-4}=0.31$), and pyrexia (${F1}_{Roche:GPT-4o}=0.33$). The overall trends in primary measures, F1 and micro-averaged F1, indicate GPT-4o as the best performing model.

**Fig. 3:**
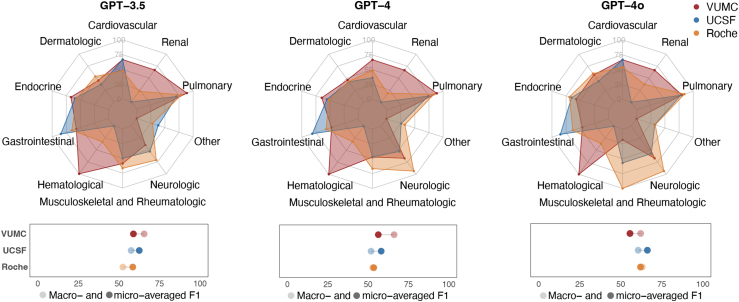
Comparative analysis of patient-level results across datasets from 3 institutions: Vanderbilt University Medical Center (VUMC), University of California San Francisco (UCSF), and Roche. The radar charts at the top highlight variations in F1 scores across irAE categories, whereas the lollipop plots at the bottom summarise the macro- and micro-averaged F1 scores. Detailed results of this analysis are provided in Table 2.

Organ-level classification of irAEs facilitates the comparison of results both across institutions and within individual irAE categories (Fig. 3 and Table 2). Across the VUMC, UCSF, and Roche datasets, the best results were observed in the haematological (F1 range = 1.0–1.0), gastrointestinal (F1 range = 0.81–0.85), and musculoskeletal/rheumatologic (F1 range = 0.67–1.0) categories, respectively. GPT models showed consistently high F1 scores in the pulmonary category across all datasets. Notably, aggregating manually annotated irAEs and irAE predictions into organ-level irAE categories improves the micro-averaged F1 scores across all datasets (Tables S9–S11).

**Table 2:** Patient-level evaluation for the identification of irAE categories across the datasets from Vanderbilt University Medical Center (VUMC), University of California San Francisco (UCSF), and Roche.

irAE Category	VUMC^a^			UCSF			Roche		
	GPT-3.5	GPT-4	GPT-4o	GPT-3.5	GPT-4	GPT-4o	GPT-3.5	GPT-4	GPT-4o
Cardiovascular	0.667	0.667	0.667	0.667	0.364	0.667	0.476	0.489	0.545
Dermatologic	0.448	0.464	0.571	0.368	0.424	0.438	0.533	0.431	0.596
Endocrine	0.667	0.647	0.571	0.596	0.556	0.652	0.571	0.525	0.689
Gastrointestinal	0.571	0.474	0.462	0.846	0.814	0.850	0.650	0.568	0.630
Haematological	1.000	1.000	1.000	NA	NA	NA	0.333	0.320	0.364
Musculoskeletal and Rheumatologic	0.588	0.471	0.182	0.500	0.467	0.571	0.667	0.667	1.000
Neurologic	0.400	0.667	0.667	0.533	0.526	0.571	0.714	0.933	0.933
Other	NA	NA	NA	0.381	0.258	0.320	0.279	0.323	0.328
Pulmonary	0.889	0.889	0.800	0.706	0.737	0.778	0.792	0.838	0.845
Renal	0.667	0.667	0.667	NA	NA	NA	0.222	0.186	0.375
**micro avg**	0.589	0.563	0.556	0.625	0.581	0.662	0.585	0.535	0.620
**macro avg**	0.655	0.661	0.621	0.575	0.518	0.606	0.524	0.528	0.631

All the result values in the table correspond to F1 scores.

a The VUMC results were extracted using the decision threshold values (i.e., the minimum number of positive predictions) corresponding to the best micro-averaged F1 scores for each GPT model.

### Note-level evaluation

A note-level evaluation was conducted to more effectively assess the robustness of GPT models, given that patient-level outcomes depend on the aggregation of irAE predictions across all notes for the same patient. Further, this type of approach facilitates the identification of irAEs over time, offering direct utility for incident-based pharmacoepidemiologic studies. Overall, despite using optimal decision thresholds for patient-level predictions, higher micro-averaged F1 scores were observed in note-level evaluation compared to patient-level evaluation for identifying both irAEs (micro-averaged F1 ranges, [0.50–0.57] vs. [0.46–0.48]) and irAE categories (micro-averaged F1 ranges, [0.61–0.66] vs. [0.56–0.59]) in the VUMC dataset (Table 3 and Table S9). Consistent with the trends shown for patient-level evaluation, GPT-4o yielded the best performing F1 scores for note-level evaluation.

**Table 3:** Note-level evaluation for the identification of irAEs and their corresponding categories in the Vanderbilt University Medical Center (VUMC) dataset.

	GPT-3.5				GPT-4				GPT-4o			
	P	R	S	F1	P	R	S	F1	P	R	S	F1
irAE												
Neuropathy	0.217	0.098	0.971	0.135	0.553	0.412	0.972	0.472	0.600	0.294	0.984	0.395
Hypothyroid	0.431	0.936	0.906	0.591	0.419	0.936	0.902	0.579	0.688	0.936	0.968	0.793
Myasthenia gravis (MG)	0.902	0.979	0.992	0.939	0.885	0.979	0.990	0.929	0.979	0.979	0.998	0.979
Rash	0.535	0.676	0.968	0.597	0.351	0.794	0.921	0.486	0.386	0.794	0.932	0.519
Colitis	0.714	0.741	0.988	0.727	0.724	0.778	0.988	0.750	0.750	0.778	0.989	0.764
Adrenal insufficiency	0.451	0.958	0.956	0.613	0.431	0.917	0.955	0.587	0.431	0.917	0.955	0.587
Hepatitis	0.750	0.750	0.991	0.750	0.655	0.792	0.984	0.717	0.633	0.792	0.983	0.704
Arthralgia	0.208	1.000	0.870	0.344	0.321	0.818	0.941	0.462	0.396	0.955	0.950	0.560
Duodenitis	0.778	0.933	0.994	0.848	0.833	1.000	0.995	0.909	0.833	1.000	0.995	0.909
Pancreatitis	0.706	0.923	0.992	0.800	0.722	1.000	0.992	0.839	0.722	1.000	0.992	0.839
Hypophysitis	0.279	1.000	0.953	0.436	0.234	0.917	0.945	0.373	0.239	0.917	0.947	0.379
Mucositis	1.000	0.583	1.000	0.737	0.526	0.833	0.986	0.645	0.818	0.750	0.997	0.783
Arthritis	0.200	0.778	0.957	0.318	0.179	0.778	0.951	0.292	0.286	0.667	0.977	0.400
Pneumonitis	0.350	0.875	0.980	0.500	0.318	0.875	0.977	0.467	0.316	0.750	0.980	0.444
Joint pain	0.037	1.000	0.883	0.071	0.067	1.000	0.937	0.125	0.045	1.000	0.904	0.086
Fever	0.111	0.667	0.976	0.190	0.071	0.667	0.961	0.129	0.080	0.667	0.965	0.143
Myalgia	0.036	0.667	0.920	0.069	0.057	0.667	0.950	0.105	0.049	0.667	0.941	0.091
micro avg	0.370	0.754	0.959	0.496	0.407	0.814	0.962	0.542	0.445	0.797	0.968	0.571
macro avg	0.453	0.798	0.959	0.510	0.432	0.833	0.962	0.521	0.485	0.815	0.968	0.551
irAE Category												
Dermatologic	0.535	0.676	0.968	0.597	0.351	0.794	0.921	0.486	0.386	0.794	0.932	0.519
Endocrine	0.580	0.945	0.916	0.719	0.535	0.945	0.899	0.683	0.793	0.945	0.970	0.862
Gastrointestinal	0.831	0.771	0.982	0.800	0.714	0.857	0.960	0.779	0.766	0.843	0.970	0.803
Musculoskeletal and Rheumatologic	0.280	0.971	0.866	0.434	0.264	0.971	0.855	0.415	0.288	0.941	0.875	0.441
Neurologic	0.859	0.561	0.984	0.679	0.791	0.694	0.968	0.739	0.847	0.622	0.981	0.718
Other	0.111	0.667	0.976	0.190	0.071	0.667	0.961	0.129	0.080	0.667	0.965	0.143
Pulmonary	0.350	0.875	0.980	0.500	0.318	0.875	0.977	0.467	0.316	0.750	0.980	0.444
micro avg	0.544	0.759	0.953	0.634	0.483	0.831	0.934	0.611	0.555	0.800	0.953	0.656
macro avg	0.507	0.781	0.953	0.560	0.435	0.829	0.934	0.528	0.497	0.795	0.953	0.562

P, precision (positive predictive value); R, recall (sensitivity); S, specificity; F1, F1-measure.

### Error analysis

The manual review of 261 notes associated with 186 (71.3%) false positive results revealed that LLMs struggle to solve causal relationships involving positively asserted events with unspecified or unrelated etiological factors; 44 (16.9%) false positive findings also included adverse events characterised by an assertion status indicating hypothetical, uncertain or possible scenarios, or events not associated with the patient. Table S12 lists examples with clinical note excerpts where such adverse events were misclassified by LLMs as irAEs. Finally, 22 (8.4%) false positives were ultimately confirmed as true irAEs, having been unnoticed during manual annotation (e.g., “*treated with ipilimumab c/b hypophysitis and colitis*”). A significant lower number of false negative results were generated by the GPT models, many of which being improperly described in the zero-shot prompt. For instance, the LLMs failed to associate terms such as “*liver inflammation*” and “*developed grade 2 elevated LFTs*” with hepatitis, as the medical concepts encoded in these expressions were missing from the hepatitis synset. Improper tokenization of specific medical terms may also impact how accurately GPT models recognise irAEs in clinical notes. Subword tokenization examples generated by Tiktoken,^37^ the tokenizer developed by OpenAI for its GPT models, include [*‘neuropathy’*] → [*‘neu’, ‘rop’, ‘athy'*], [*‘neurotox’*] → [*‘ne’, ‘uro’, ‘to’, ‘x’*], [‘*myasthenia gravis*’] → [*‘my’, ‘ast’, ‘hen’, ‘ia’, ' gr’, ‘avis’*], and [‘*hypothyroid’*] → [*‘h’, ‘yp’, ‘othy’, ‘roid’*].

## Discussion

The primary objective of this study was to establish irAE-GPT as a high-throughput, generalisable system for the automatic identification of therapy-associated toxicities in clinical text. GPT models demonstrated the potential to effectively identify immune-related adverse events (irAEs) in two distinct textual modalities: clinical notes from two large, tertiary, real-world EHR systems, as well as patient reports from seven clinical trials. Moreover, the models generalised across diverse clinical contexts: VUMC included both inpatient and outpatient settings across all irAE severities; UCSF focused on hospitalised patients with severe irAEs; and Roche evaluated patients in clinical trials who experienced at least one irAE.

The closest study to ours is by Sun et al.,^12^ where they employed the open-source LLM Mistral OpenOrca^38^ to detect four irAEs (colitis, hepatitis, myocarditis, and pneumonitis) in electronic health records of hospitalised patients at Massachusetts General Hospital (MGH) and the Brigham and Women's Hospital (BWH). A straightforward analysis using averaged F1 scores over the four irAEs revealed that Mistral OpenOrca underperformed on the MGH (mean F1 = 0.26) and BWH (mean F1 = 0.29) datasets compared to GPT models, which performed better on the VUMC (mean F1 = 0.60), UCSF (mean F1 = 0.73), and Roche (mean F1 = 0.65) datasets. Here, the Roche results reflect the averaged F1 scores for identifying colitis, hepatitis, and pneumonitis, as myocarditis events were absent in the corresponding dataset. Given the indirect nature of this comparison between GPT models and an open-source LLM, future investigations utilising a broader range of open-source models or models pre-trained on biomedical or clinical corpora, such as Llama,^39^^,^^40^ BioGPT,^41^ or Med-PaLM,^42^ may offer deeper insights into irAE identification.

With the anticipated release of new ICD-10 codes for ICI-associated irAEs,^13^ the classification of irAEs is expected to become more precise, leading to improvements in data availability, comparability, and quality. However, the consistent and routine adoption of these new codes by clinicians, as well as their validation in clinical applications, will require time. The synergy between irAE-GPT and the new ICD codes could enable a systematic approach to understanding the spectrum and burden of ICI-associated irAEs, with far-reaching implications for clinical practice, public health, and research.

The evaluation of the GPT models revealed several significant findings, reinforcing the methodological and analytical strengths of the study. First, the models achieved consistently high recall/sensitivity and specificity values at the cost of moderate precision scores. This resulted in significantly more false positives than false negatives, indicating a bias in the GPT models toward sensitivity or overpredicting positive outcomes. One of our experiments to mitigate this bias focused on adjusting the decision threshold to optimise the F1 score, achieving a more balanced trade-off between precision and recall. Nevertheless, the results indicate that irAE-GPT can effectively facilitate pharmacoepidemiologic studies by guiding the identification of irAE cases at scale and alleviating the workload of physicians and healthcare professionals who are often responsible for manually curating such adverse events. However, the relatively high false positive rate limits its current suitability for real-world deployment to identify and characterise irAEs in patients receiving ICI therapies. Second, differences in performance were noted among the various types of evaluations conducted. The note-level evaluation yielded better results than the patient-level evaluation, possibly influenced by how note-level predictions were combined to derive patient-level outcomes. Moreover, the prediction of irAE categories demonstrated higher performance values compared to specific irAE evaluation results, primarily due to the relaxed approach in positively predicting the organ-level adverse events. For instance, positively predicting any one of pneumonitis, wheezing, influenza-like symptoms, ARDS, or pleuritis is sufficient to classify the pulmonary category as positive. Finally, the error analysis highlights the challenges faced by LLMs in handling textual causation, a task more complex than named entity recognition, where medical conditions identified in clinical text (e.g., adverse events) must also be linked to specific etiological factors (e.g., immune checkpoint inhibitors).

Our study also has several limitations that warrant further investigation in future research. First, we did not investigate widely-used methods for enhancing the performance of LLMs, including prompt engineering, few-shot learning, and fine-tuning.^16^^,^^35^^,^^43^ For example, our groups and others have previously demonstrated that LLMs are highly sensitive to the way prompts are constructed, and that prompt augmentation and optimisation play a key role in improving the performance of various tasks.^21^^,^^34^, ^35^, ^36^ Second, given that GPT models cannot feasibly process all notes from a patient within a single context window (e.g., gpt-35-turbo-16k supports only 16,384 input tokens), we employed the patient-level approach in which the LLM analysed notes sequentially, followed by aggregation of irAE predictions across all patient notes. Even models with larger context windows cannot accommodate the volume of notes observed in real-world datasets. For example, VUMC patients average 264 notes during the ICI exposure period. Although our strategy overcomes context-window constraints, it introduces additional complexity via a tunable parameter required to establish an optimal decision threshold. Our exploratory analysis characterises how LLM performance varies across the parameter's potential range. In future work, we intend to use retrieval-augmented generation (RAG) techniques^44^ as an alternative approach, creating one prompt per patient that contains only irAE-relevant text snippets extracted from the entire patient record. Third, although the study aimed to comprehensively evaluate irAE identification using GPT models across events of varying frequency and severity, results for rare irAEs should be interpreted with caution due to their susceptibility to substantial bias and the limited statistical robustness and generalisability inherent to low-prevalence outcomes. Fourth, the possibility that LLMs may perpetuate and exacerbate health disparities in clinical studies of cancer immunotherapy safety and effectiveness has not yet been explored. While not within the scope of our study, the implementation of fairness assessments and bias mitigation techniques is critical for ensuring transparent and equitable LLM approaches in such studies. Prior research has highlighted the risk of LLMs perpetuating societal biases, potentially leading to harm across patient groups.^45^, ^46^, ^47^, ^48^ In the context of irAEs, biased GPT models may disproportionately misclassify patients from certain groups, resulting in their underrepresentation in pharmacoepidemiologic research and delayed identification in pharmacovigilance efforts, which could potentially compromise timely care and effective management. Fifth, although this study focused on evaluating irAE-GPT as a generalisable, high-throughput system across diverse data modalities, clinical contexts, and irAE types, our use of the same zero-shot prompt template for all irAEs limited our ability to conduct irAE-specific analyses tied to diagnostic certainty. To address this, future extensions should integrate irAE-specific features derived from multiple data types including structured data elements (e.g., elevated TSH levels, BAL findings) to improve identification performance. Lastly, a central limitation of our approach is the intrinsic diagnostic uncertainty associated with many irAEs. In the absence of gold-standard diagnostic tests for most immune-related toxicities, expert clinical attribution remains the de facto reference standard. As a result, the binary labels in our dataset, though grounded in expert judgement, inevitably reduce a spectrum of diagnostic confidence to dichotomous categories. Future versions of irAE-GPT may benefit from incorporating continuous or probabilistic outputs to better reflect the nuanced diagnostic landscape of irAEs. This limitation is further exacerbated by the absence of a unified, comprehensive taxonomy for irAEs and by significant variability across institutions in how individual toxicities are defined, subdivided, or aggregated, factors that may limit the applicability of our findings to other institutions or treatment centers.

In conclusion, this study introduces irAE-GPT, a high-throughput phenotyping system that leverages GPT models to systematically identify irAEs in unstructured patient notes from multiple EHR systems and clinical trials. The GPT models demonstrated robust performance and generalisable capabilities in identifying irAEs at both patient and note levels as well as across varied healthcare datasets and clinical contexts, reducing the need for manual annotation of adverse events in clinical text and highlighting their potential to improve the characterisation of the safety profile of cancer immunotherapies. Efforts should continue to enhance the capabilities of these models in identifying textual causation between medication exposures and adverse events, while also addressing their potential biases in extraction of safety data from clinical text. irAE-GPT is publicly available to facilitate collaborative benchmarking across diverse healthcare datasets and to promote research reproducibility and transparency.

## Contributors

CAB, MW, SV, DBJ, EJP, JMB, RM, and ZQ contributed to the conception and design of the study. CAB, MW, and SV performed data analysis, model development, and validation. DBJ and ZQ led the manual chart review efforts on EHR data. EAB provided administrative support. CAB, MW, SV, LH, YX, JS, FW, KS, DK, EM, MSK, LY, DBJ, JMB, RM, and ZQ helped with the collection and assembly of data. CAB, MW, SV, EAB, GSC, SB, DBJ, EJP, JMB, RM, and ZQ participated in the analysis and interpretation of the results. CAB, MW, and SV contributed equally to the study. JMB, RM, and ZQ provided joint supervision. All authors were involved in drafting and revising the manuscript, approved the final version, and agreed to the decision to submit for publication. CAB, MW, and SV accessed and verified the data supporting the findings of this study.

## Data sharing statement

The source code used in the analysis is publicly available on GitHub at https://github.com/bejanlab/irAE-GPT.git. The summary statistics extracted for this study are provided in the manuscript and supplementary material. Any request to access the Synthetic Derivative data will need to be reviewed and approved by Vanderbilt University Medical Center. Researchers will need to provide evidence of IRB approval for their study. For the approved studies, data will be released via a Data Use Agreement.

## Declaration of interests

DBJ has served on advisory boards or as a consultant for AstraZeneca, BMS, The Jackson Laboratory, Merck, Mosaic ImmunoEngineering, Novartis, Pfizer, and Teiko, and has received research funding from BMS and Incyte, and has patents pending for use of MHC-II as a biomarker for immune checkpoint inhibitor response, and abatacept as treatment for immune-related adverse events. MW has received research support from the U.S. Food and Drug Administration (FDA), Amgen, BeiGene, and Merck. SB received honorarium for lecture from Merck and served as an expert witness for Colling Gilbert Wright & Carter, Wilcoxen Callaham LLP, Lee Hunt Esq, Larson Health Advocates. EJP has served as a consultant for Janssen, Rapt, Servier, Espirion, Verve, Elion and UpToDate outside of the submitted work, and receives royalties from UpToDate. EAB, GSC, and RM are employees and shareholders of Roche. They received support for preparation of this manuscript and are co-inventors on patents filed by Roche related to use of atezolizumab. ZQ has consulted for Novartis and Sanofi.
